# Radiogenomics: Current Understandings and Future Perspectives

**DOI:** 10.1002/mco2.70583

**Published:** 2026-01-22

**Authors:** Xinyu Zhang, Qingpei Lai, Jin Cao, Jerry Chi Fung Ching, Xinzhi Teng, Jiang Zhang, Shara Wee Yee Lee, Ge Ren, Jing Cai

**Affiliations:** ^1^ Department of Health Technology and Informatics The Hong Kong Polytechnic University Hong Kong China; ^2^ The Hong Kong Polytechnic University Shenzhen Research Institute Shenzhen China

**Keywords:** artificial intelligence, multiomics, precision medicine, radiogenomics

## Abstract

Radiogenomics is a rapidly developing field that links radiological image features (radiomics) to genomic‐level data (genomics, transcriptomics, and epigenomics), addressing the limitations of single‐omic approaches. Radiomics provides a noninvasive and cost‐effective method to capture tissue‐level characteristics, while genomics elucidates the underlying molecular mechanisms. The central hypothesis is that the formation of imaging phenotypes is associated with the genetic and molecular processes, and thus can reflect underlying biological activities. This review presents the fundamental principles of radiogenomic analysis, covering key concepts in image analysis and gene analysis, as well as advanced analytical techniques for linking imaging and genomic data. Moreover, we summarize recent research findings across various human diseases, including oncology and nononcology, to highlight the current understandings and achievements in this field. Radiogenomics shows potential in clinical applications for elucidating disease mechanisms, detecting genomic variations noninvasively, and improving prognosis predictions. However, its implementation in clinical practice is limited by data scarcity, analytical methods, and barriers in translational processes. Future research should focus on enhancing data quality and establishing guidelines, developing analytical platforms, and validating current findings through animal models and clinical trials.

## Graphical abstract

Radiogenomics links imaging phenotypes with genetic variations, offering potential for comprehensive understanding, cost‐effective diagnosis, and prognosis prediction to advance personalized medicine. However, its clinical application remains limited by several challenges. Further research is needed to establish guidelines and platforms and validate the findings in animal experiments and clinical trials.

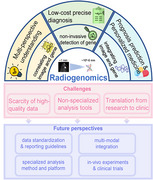

## 1 Introduction

Radiogenomics is an emerging interdisciplinary field that integrates radiomics and genomics. As the intersection of two omics fields, the history of radiogenomics can be traced to the origin of omics. The concept of “omics” emerged in the early 1990s with the initiation of the Human Genome Project, which marked a significant milestone in the systematic exploration of human diseases at the molecular level and gave rise to the field of human genomics. As research progressed, the spectrum of omics rapidly expanded beyond genomics to encompass fields such as proteomics and metabolomics [1]. This expansion has enabled a more holistic understanding of human diseases by capturing diverse molecular activities and interactions. The rapid advancement of omics technologies has brought revolutionary breakthroughs to precision medicine. Traditionally, these omics disciplines have focused on the activities and alterations of small molecules, representing the microlevel variations underlying disease processes that are closely related to the clinical management of diseases. However, the clinical implementation of traditional omics has been limited by several factors, including the need for tissue sampling, the high cost and time‐consuming nature of genomic tests, and the inherent bias introduced by tissue heterogeneity.

Building upon the foundation established by traditional omics, radiomics was introduced by Lambin et al. in 2012 as a novel approach to disease characterization and quickly became an important component in the omics spectrum [2, 3]. Unlike conventional omics that analyze molecular data, radiomics involves the extraction of abundant quantitative features from medical images, thereby capturing macrolevel variations in tissue and organ structure. This imaging‐based perspective complements molecular analysis and offers additional perspective on disease phenotypes. Radiomics is inherently objective, noninvasive, and cost effective, making it an optimal choice for routine use in clinical practice. Furthermore, radiological images can overcome the issue of tissue heterogeneity by providing comprehensive coverage of the entire target regions without additional sampling cost.

The convergence of genomics and radiomics has led to the emergence of radiogenomics. In this context, radiomics refer to the features extracted from routinely used radiological images in clinical practice, while genomics encompass data from DNA sequences (genomics), gene expression regulators (epigenomics), and gene expression products (transcriptomics) [4]. The fundamental hypothesis in radiogenomics is that imaging phenotypes are the manifestations of variations in underlying biological processes. By correlating and combining multiscale information, it enables simultaneous exploration of diseases at both the molecular and imaging phenotypic levels. It is worth noted that the term “radiogenomics” is also widely used in studies investigating the association between gene expression and radiotherapy effect, which is beyond the scope of this review and thus not included.

Recent advances in radiogenomics have yielded a series of clinically enlightening findings, such as improved diagnostic accuracy, refined disease subtyping, enhanced prognostic prediction, and a deeper understanding of imaging–genetic associations across a broad spectrum of human diseases [5, 6, 7, 8, 9, 10]. The growing body of radiogenomics research reflects the strong academic and clinical interest in leveraging radiogenomic approaches to address key challenges in human disease management. The purpose of this review is to systematically summarize recent key advances in the field across various human diseases. In addition, this review discusses the potential clinical implications of these findings, providing a comprehensive reference for future research and the translation of radiogenomic insights into clinical practice.

The literature search and selection processes are shown in Figure S1. The remainder of this review is structured as follows. In Section 2, the fundamental principles of radiogenomic analysis are introduced, including basic information regarding radiological image and genomic data analysis as well as principles of radiogenomic analysis. In Section 3, we introduce the techniques used in radiogenomic analysis, covering basic statistical analysis and sophisticated artificial intelligence methods. In Section 4, recent radiogenomic studies published are summarized according to disease type, which generally divide into cancer and noncancer diseases. In Section 5, an in‐depth discussion about the clinical implications, existing challenges, and future perspectives of radiogenomics is given.

## 2 Foundations of Radiogenomics

Radiogenomics refers to the integrative analysis of radiological image and gene information. In this section, we first offer an overview of the characteristics and representative techniques associated with radiological images and gene information respectively, encompassing their raw data acquisition, initial processing, and advanced analytical methods. In the last section, we outline the fundamental principles and key challenges inherent in the integrative analysis of imaging and genomic data.

### 2.1 Principles of Radiological Imaging and Radiomic Analysis

**FIGURE 1:**
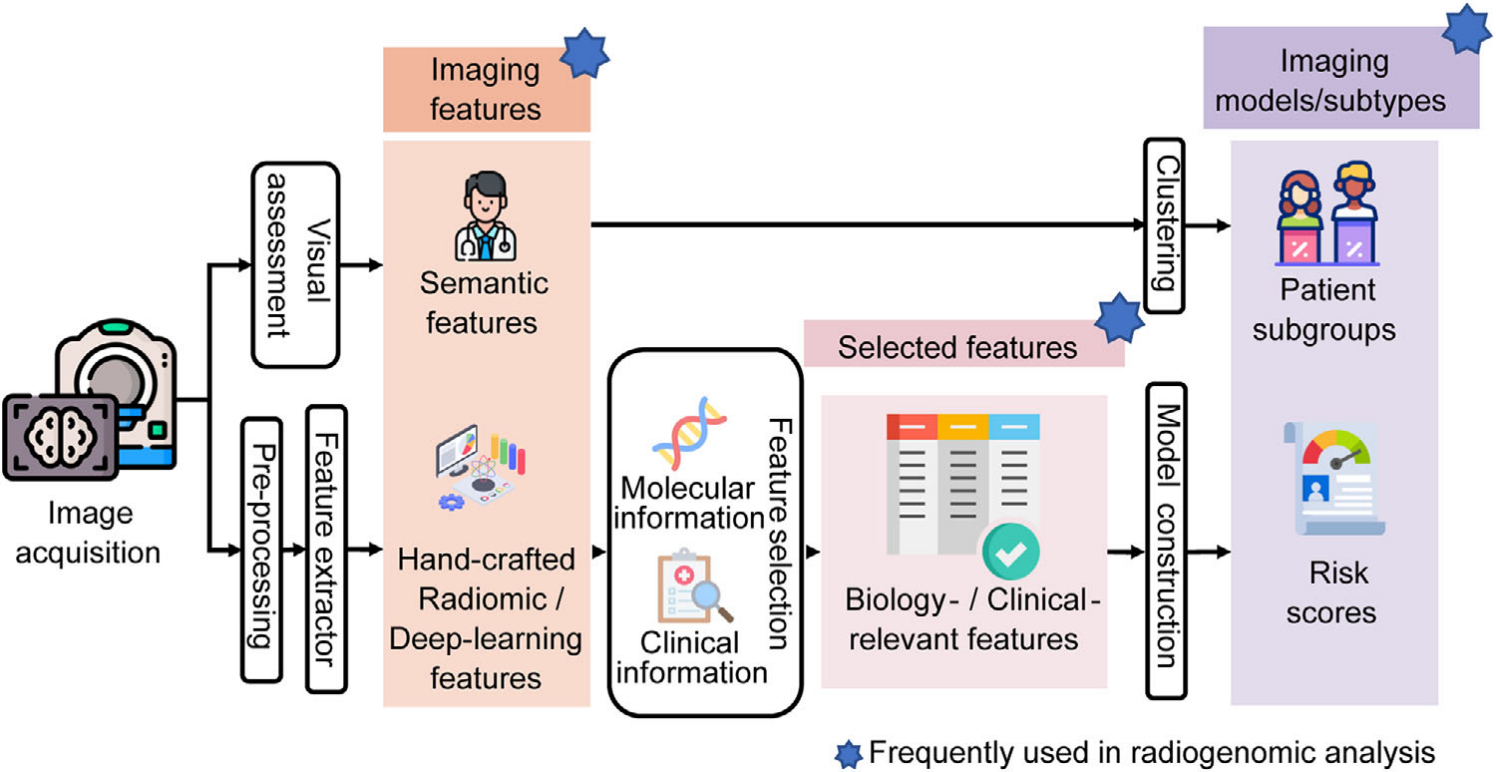
Typical workflow of radiological image analysis for personalized medicine. The process begins with image acquisition, followed by features extraction through two branches: (1) visual assessment to obtain semantic features; and (2) computational processing to extract hand‐crafted and deep learning features. The extracted features are either directly clustered to obtain imaging subtypes or undergo a series of feature selection and model construction to develop imaging models. The radiogenomic analysis can be performed the initially extracted features, the selected features with specific clinical or biological relevance, or the identified models/subtypes. (This figure has been produced using resources from Flaticon.com.)

Multimodal medical imaging serving as biomarkers has been the cornerstone for precision medicine, depicting patients’ biological status from the anatomical or functional aspects. Common two‐dimensional imaging modalities include X‐ray radiograph, mammography, and ultrasound. Volumetric imaging includes computed tomography (CT), magnetic resonance imaging (MRI). Functional imaging includes positron emission tomography (PET), single‐photon emission computed tomography. Each modality provides unique anatomical or functional data to facilitate personalized medicine. General X‐ray examinations enable rapid, high‐throughput evaluation; CT enables the volumetric representations of anatomy; MRI offers enhanced soft‐tissue contrast and multiparametric characterization; PET provides quantitative insights into metabolism that improve risk stratification [11, 12, 13, 14]. Applications of such span across the entire care continuum, such as screening, diagnosis, management planning, real‐time treatment guidance, and posttreatment follow‐up [15, 16]. Currently, image‐guided procedures such as interventional radiology, targeted biopsies, and radiotherapy have been the standard of care. This leads to the emergence of large, raw, and multimodal imaging datasets that are available throughout the path of clinical management [17, 18]. The increase in high‐fidelity data opens new possibilities for quantitative analysis and decision support, setting the stage for data‐driven precision medicine (Figure 1).

Image phenotypes, or more commonly known as image features, extracted can be broadly categorized as semantic and quantitative, algorithmically derived descriptors. Semantic features are human‐interpretable attributes defined within standardized lexicons. Widely recognized semantic features reporting systems include Breast Imaging Reporting & Data System for breast imaging, Prostate Imaging Reporting & Data System for prostate MRI, and Liver Imaging Reporting & Data System for liver lesions [19, 20, 21]. Reporting systems encode expert assessments of morphology, margins, and overall abstract impression of the disease. However, such impression could be subjective in nature, prone to interviewer variability.

Quantitative features, often termed radiomic features, are objective measurement of image phenotypes computed via algorithms that summarize shape, intensity distributions, texture, and higher‐order patterns within regions of interest (ROIs) [2]. Radiomics is the systematic high‐throughput extraction of quantitative image features to develop predictive or prognostic models that enable personalized oncology [22, 23]. Image preprocessing such as intensity normalization and binning, voxel resampling, and filtering are usually performed before feature extraction. The Image Biomarker Standardisation Initiative (IBSI) and its subsequent publication has provided rigorous guidelines on practical knowhow and definitions for these hand‐crafted radiomic features, improving their reproducibility across software implementations and centers [24, 25]. Deep learning (DL) radiomic features, learned by convolutional and transformer‐based networks are increasingly prevalent. Such features can provide high‐dimensional abstractions without manual definition by propagating gradients from task labels, thereby discovering hierarchical filters and embeddings that capture complex phenotypes [26, 27, 28]. Despite promising gains in detection, risk stratification, and outcome prediction, these models remain partially “black‐box,” with open challenges in interpretability and attribution. Together, semantic and quantitative radiomic features enable multiscale, multimodal phenotyping that complements clinical workup.

Currently, radiomics mainly contributes to disease detection, risk stratification, treatment response prediction, survival prediction, and toxicity prediction [29, 30, 31, 32]. For instance, by extracting characteristics from serial pretreatment images, researchers can pinpoint subregions demonstrating early radiographic change and guide adaptive replanning tactics [32, 33]. Apart from oncology, radiomics can also be applied to other clinical fields. In neurology, different radiomic models have been built for the identification and prognosis of neurologic disorders, such as Alzheimer's disease [34], ischemic stroke [35], intracranial hemorrhage [36], Parkinson's disease [37], and schizophrenia [38]. Occasional uses of radiomics are also seen in cardiology for the analysis of vascular diseases [39].

Recent progress in radiomics emphasize methodological rigor. Research have been pushing toward standardizing acquisition and reporting with the introduction of Radiomics Quality Score 2.0 (RQS) and CheckList for EvaluAtion of Radiomics Research (CLEAR) to codify radiomics best practice [40, 41]. Multicentre external validation, calibration, and decision‐curve analysis have been suggested to curb bias, preventing the development of overfitting model and thus enhancing clinical utility [25]. IBSI‐compliant feature definitions, and explicit assessment of feature stability are also expected to improve reproducibility and applicability in future radiomic projects [42]. Building on these foundations, disease‐specific guidance has emerged, the Prostate cancer patient characteristics reporting items (PCPCRI) specify minimal reporting items [43]. Disease‐specific guidance is expected to further improve validity of future radiomic projects. The added robustness can facilitate cross‐study synthesis and clinical interpretability of valuable research [44, 45].

### 2.2 Principles of Genomic Profiling

**FIGURE 2:**
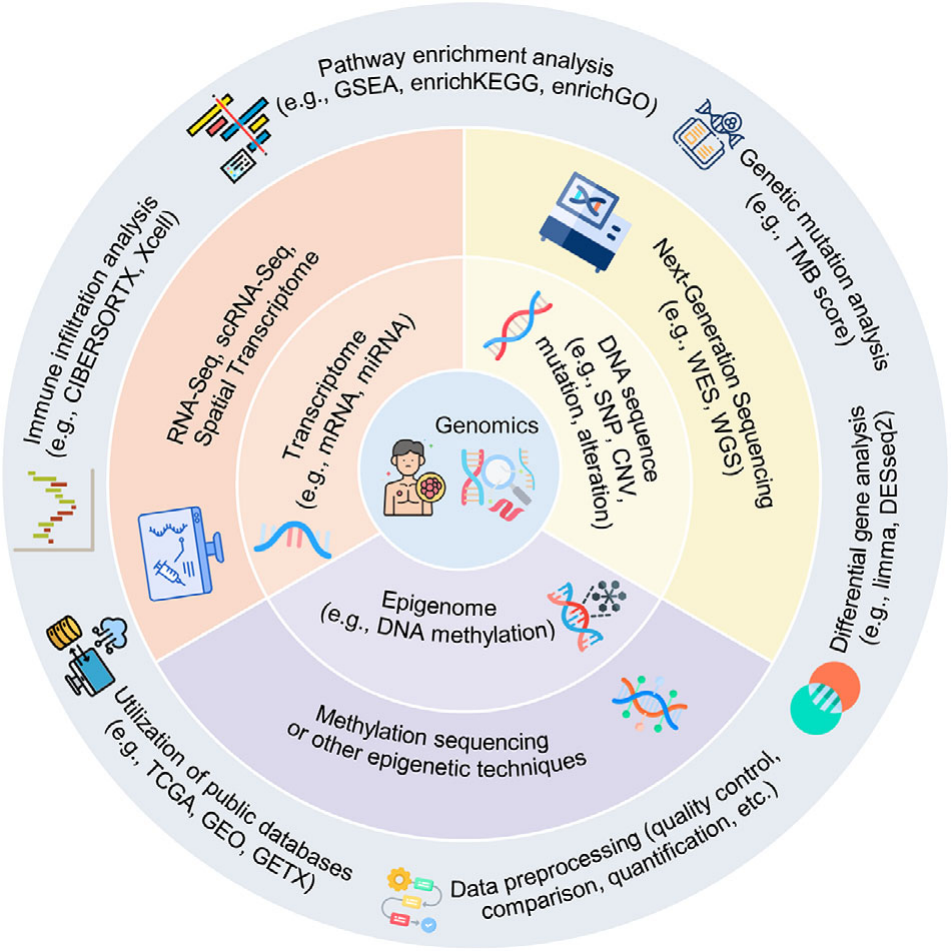
Summary for genomic profiling and related techniques. This circular diagram illustrates the broad categories of genomics (transcriptomics, DNA, and epigenomics) in the inner ring, the corresponding acquisition technologies (such as RNA sequencing, next‐generation sequencing, and methylation/epigenomic sequencing) in the middle ring, and the outer ring highlights key analysis methods (e.g., data cleaning, immune cell infiltration estimation, pathway, and mutation analysis) and major data storage resources (such as TCGA and GEO). (This figure has been produced using resources from Flaticon.com.)

Genomic profiling offers unprecedented depth of insight into disease diagnosis, prognostic assessment, and therapeutic decision‐making by systematically identifying and quantifying molecular information within biological systems [46]. Broadly defined, genomic data encompass not only nuclear DNA sequence information, such as single nucleotide polymorphisms (SNPs) and copy number variations that directly reflect oncogenic mutations and structural alterations, but also transcriptomic data that reflect gene expression levels and thus the dynamic activity of genes under specific temporal and environmental condition [47, 48]. Furthermore, epigenomic information, particularly DNA methylation status, has emerged as a critical regulatory mechanism, increasingly recognized as an essential component for elucidating gene expression differences and phenotypic variability [49]. Figure 2 shows a summary of various genomic information and related techniques.

Acquisition of such vast and multilayered genomic data relies fundamentally on advanced technologies. Among these, next‐generation sequencing stands as a methodological core, revolutionizing traditional sequencing paradigms by enabling researchers to perform whole‐exome sequencing or whole‐genome sequencing in a cost‐effective and high‐throughput manner [50]. This facilitates the comprehensive identification of all potential genetic variations within a given sample. For transcriptomic profiling, RNA sequencing technology not only allows for the precise quantification of mRNA abundance but also enables regulation [51, 52].

In recent years, the advent of single‐cell sequencing technologies has further advanced the field by enabling the dissection of cellular heterogeneity within tumors at an unprecedented resolution [53, 54]. Single‐cell RNA sequencing allows for the characterization of gene expression profiles at the individual cell level, uncovering rare cell populations and dynamic cellular states that are often concealed in bulk analyses [54, 55]. This is particularly valuable in oncology, where intratumoral heterogeneity significantly influences disease progression and therapeutic response. Complementing this, spatial transcriptomics technologies have emerged, which not only quantify gene expression but also preserve the spatial context of cells within tissue architecture [56]. By integrating spatial information, researchers can map the localization of distinct cell types and gene expression patterns within the tumor microenvironment (TME), providing critical insights into the cell‐cell interaction and spatial organization of molecular signals relevant to radiotherapy outcomes [57].

The transformation of raw sequencing data into clinically actionable insights relies heavily on robust data analysis and bioinformatics methodologies. The analytical workflow typically begins with rigorous preprocessing steps, including quality control, alignment to reference genomes, and quantification of molecular features. Subsequent analyses often involve differential gene expression analysis to identify genes that are significantly altered between diseases and control states, providing key molecular signatures relevant to diagnosis and prognosis [58]. Pathway enrichment analyses, such as Gene Set Enrichment Analysis (GSEA), further contextualize these findings by highlighting biological pathways and processes that are dysregulated in specific disease contexts [59]. The integration and interpretation of genomic data are greatly facilitated by public repositories such as The Cancer Genome Atlas (TCGA) and the Genotype‐Tissue Expression project, which provide comprehensive, standardized datasets for comparative analyses and validation of findings. These resources enable researchers to benchmark their results, explore population‐level genetic variation, and identify clinically relevant biomarkers. In the realm of tumor immunology, computational tools such as CIBERSORT [60, 61] and xCell [62] are employed to infer the abundance of various immune cell types from bulk gene expression data, offering insights into the TME and its impact on therapeutic response.

These cutting‐edge technologies, when integrated with sophisticated bioinformatics pipelines, ultimately enable the transformation of raw molecular signals into actionable molecular maps that can inform and guide clinical decision‐making. Moreover, the continuous advancement of sequencing platforms and analytical algorithms further enhances the resolution and interpretability of genomic data, paving the way for more personalized and effective therapeutic strategies in oncology and beyond.

### 2.3 Principles of Radiogenomics

The foundation of radiogenomics lies on a key hypothesis: the biological and molecular changes underlying diseases, such as gene expression, mutations, and metabolic processes, are reflected in medical images through changes in tissue morphology, density, and blood supply. Medical images, therefore, are not merely representations of tissue anatomical structure, but the result of complex genetic and molecular interactions and inherently contain information about the genome, transcriptome, and other molecular activities. Imaging and gene data each provide different yet interrelated perspectives on disease status, offering a complementary view of underlying mechanisms. The main challenge in radiogenomics is to establish meaningful connections between imaging features and genomic/molecular data, enabling the integration and mutual interpretation of these two information sources. Ultimately, this field aims to support a deeper understanding of disease mechanisms and enhance clinical decision‐making.

## 3 Advanced Techniques in Radiogenomics

This section provides a comprehensive overview of the advanced techniques commonly used in radiogenomic studies, including fundamental statistical methods as well as sophisticated artificial intelligence approaches. Each category of these methods offers unique advantages and perspectives for the analysis of radiogenomic associations.

### 3.1 Statistical Approaches

**FIGURE 3:**
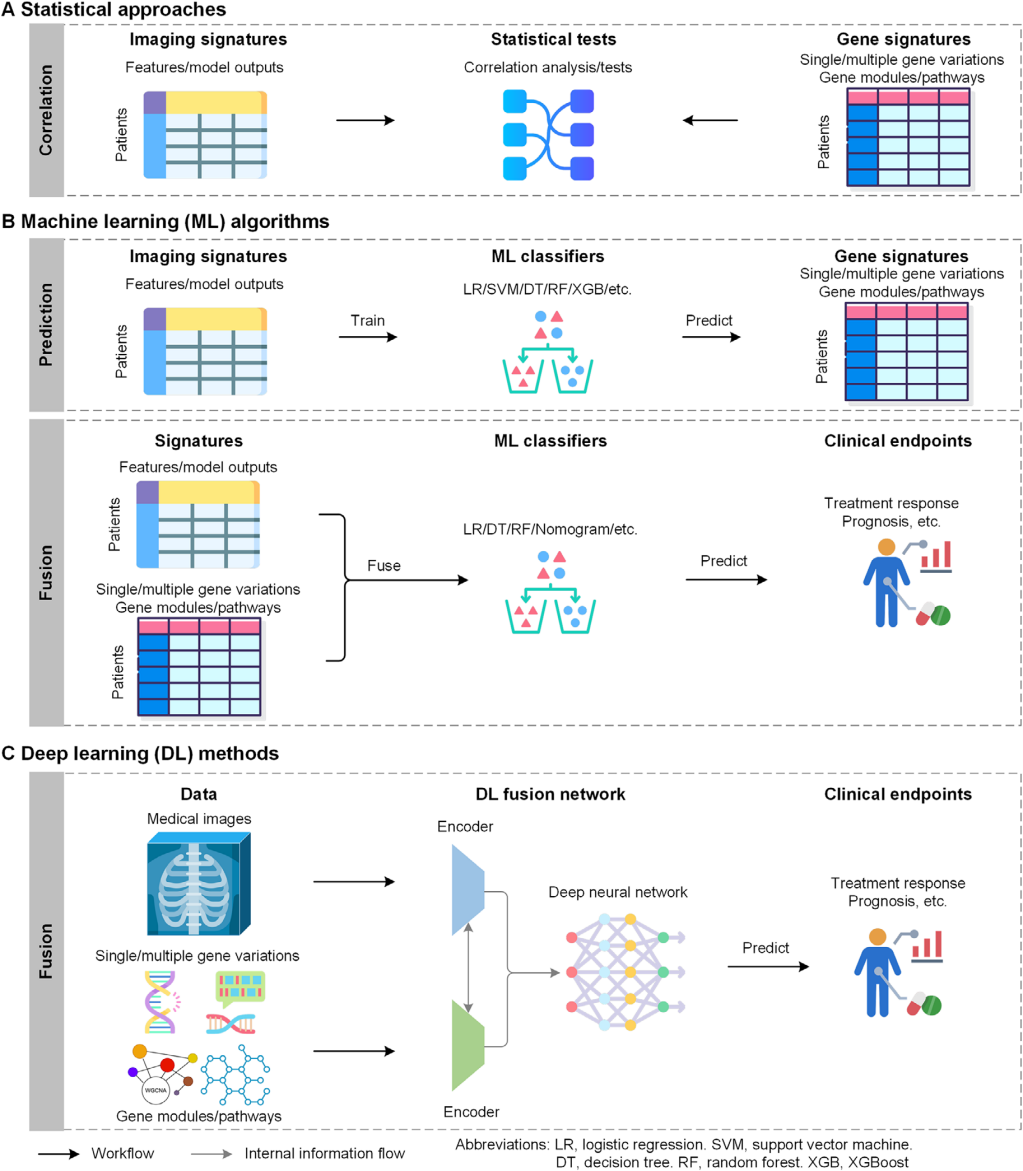
Overview of advanced techniques in radiogenomics. The figure illustrates three primary tec for integrating imaging and genomic data. (A) Statistical approaches are typically used to establish correlations between imaging signatures and gene signatures. (B) Machine learning (ML) algorithms can be used to predict gene signatures from imaging features or to fuse both data types to predict clinical endpoints. (C) Deep learning networks enable end‐to‐end fusion of raw medical images and various forms of genomic data for predicting clinical outcomes. (This figure has been produced using resources from Flaticon.com.)

Establishing correlations between imaging features and single gene status is driven by a critical clinical need. Many genes exhibit significant associations with clinical endpoints such as antitumor treatment response and survival. With this regard, statistical correlation tests are widely employed to analyze differences in imaging features between patients with different gene expression or mutation status (Figure 3). This identifies the intrinsic or direct associations between imaging features and the status of the target gene. Commonly used methods include Pearson correlation analysis, Spearman correlation analysis, Fisher's exact test, Pearson's Chi‐square test, the Wilcoxon rank‐sum test, and so on (Table 1). For example, Cui et al. used Pearson correlation coefficient to evaluate the linear correlation between radiomic features and gene expressions (both continuous data) [63], while Greco et al. employed Pearson's Chi‐square test to evaluate the statistical significance of proportional differences in binary data (e.g., patient gender, tumor stage) among patient cohorts stratified by NCOA7 expression [64].

**TABLE 1:** A summary of common statistical, machine learning (ML), and deep learning (DL) approaches in radiogenomics.

Method name	Category	Method purpose	Data requirement	References^a^
Spearman correlation analysis	Statistical	Correlation	Tabular features	[65, 66, 67, 68, 69]
Pearson correlation analysis	Statistical	Correlation	Tabular features	[63, 70, 71]
Pearson's Chi‐square test	Statistical	Correlation	Categorical variables (e.g., tumor stage, risk stratification)	[64, 72, 73, 74]
Wilcoxon rank sum test	Statistical	Correlation	Tabular features	[72, 73, 74]
Student's *t*‐test	Statistical	Correlation	Tabular features	[64]
Mann–Whitney *U* test	Statistical	Correlation	Tabular features	[75, 76]
LASSO	ML	Prediction	Tabular features	[77, 78, 79]
GBM/lightGBM	ML	Prediction	Tabular features	[80, 81, 82, 83]
XGBoost	ML	Prediction	Tabular features	[84, 85, 86]
Decision tree	ML	Prediction/fusion	Tabular features	[84, 86]
Logistic regression	ML	Prediction/fusion	Tabular features	[77, 87, 88, 89]
Random forest	ML	Prediction/fusion	Tabular features	[75, 84, 85, 86, 90]
Support vector machine	ML	Prediction/fusion	Tabular features	[75, 84, 85]
Nomogram	ML	Fusion	Tabular features	[91, 92, 93, 94, 95, 96]
Self‐and‐mutual attention	DL	Fusion	CT image data and RNA‐seq data	[97]
Multiview nonnegative matrix factorization	DL	Fusion	DCE‐MRI data, DNA copy number alterations, mutations, and mRNAs	[98]
Regularized adaptive sparse multiset canonical correlation analysis	DL	Fusion	12 multiangle ultrasound images and gene mutation data	[99]

^a^ References listed some examples of studies employing the corresponding analysis approach.

However, single‐gene analysis is often insufficient as genes typically function in networks rather than in isolation. With recent development of sequencing techniques, statistical approaches have expanded to explore the associations between imaging features and the entire gene expression profile or with gene modules obtained by methods like weighted gene coexpression network analysis [91, 100, 101]. Subsequently, GSEA is performed to identify the functional pathways enriched among the correlated genes or modules, providing insights into their combined functions [79, 102, 103]. Studies have found the associations between prognosis‐related imaging features or models with cell cycle and immune pathways that significantly influence tumor aggressiveness [72, 74, 104]. Su et al. validated the upregulation of Ferroptosis pathway in patients exhibiting higher imaging intratumor heterogeneity, implying the potential therapeutic target for this specific patient group [105]. These findings highlight the significance of large‐scale correlation analys is and pathway analysis over single‐gene correlation analysis.

### 3.2 Machine Learning Algorithms

In addition to traditional statistical approaches, machine learning (ML) algorithms have been increasingly employed in radiogenomics for both gene status prediction and multimodal data fusion.

First, for predicting gene status, algorithms aim to build predictive models from imaging features (independent variables) to predict gene expression or mutation status (dependent variable) (Figure 3). Commonly used models include classical ML algorithms such as support vector machine (SVM) [66, 67, 68, 106], XGBoost [9, 66, 85, 107], logistic regression [108, 109, 110, 111], random forest [86, 90, 112, 113], and decision trees [66, 76, 114, 115] (Table 1). These methods are selected not only for their predictive performance but also for generalizability, reliability, and interpretability. Meanwhile, given the high dimensionality of imaging features, especially radiomics and deep‐learning features, feature selection is usually performed prior to model training to reduce dimensionality and prevent severe overfitting [9, 110, 115]. Studies have employed various feature selection methods and models and compared their performance using metrics like area under the receiver operating curve (AUC), accuracy (ACC), sensitivity (SEN), specificity (SPE), and so on [110, 116, 117].

Second, ML facilitates “phenotype‐molecule” synergy by fusing radiomic and genomic information (Figure 3). The most straightforward approach involves preprocessing the features separately, then concatenating them as inputs for model training [118, 119, 120]. Beyond direct feature‐level fusion, some studies use filtered genomic features and the Radscore output by radiomic models as inputs for logistic regression to predict disease‐free survival [77, 87, 88, 89]. Nomogram, a popular linear regression method, are also widely used to combine radiomics and immune‐related genes signatures for predicting the risk of metastasis [91, 92, 93, 94, 95]. Other traditional ML methods like random forest [75], SVM [75], and decision tree [89] possess nonlinear fitting capabilities and thus can explore distinct information inherent to radiomics and genomics.

### 3.3 DL Networks

While ML offers linear, interpretable feature fusion, DL enables high‐dimensional, nonlinear fusion in the potential space, fully exploiting inherent complementary predictive information between radiomics and genomics (Table 1). Assigning distinct encoders to different modal inputs with intermediate‐layer fusion is a widely adopted, easily deployable radiogenomics approach [7, 121]. However, some studies argue that encoders alone cannot fully harness modality‐specific information, suggesting separate modules for imaging, genomics, and fusion to extract and integrate features [122].

Notably, more innovative architectures have emerged for fusing imaging and genomic data. Kawahara et al. used t‐SNE to map selected preprocessed radiomic/genomic eigenvector features to images and feed them into the CNN‐based DeepInsight for disease‐free survival prediction [123]. There are other interesting networks such as self‐and‐mutual attention network [97], and semi‐supervised models like the semi‐supervised multimodal multiscale attention model [124]. Collectively, these multimodal strategies, from simple encoder‐based methods to innovative attention/semi‐supervised architectures, leverage imaging‐genomic complementarity to robustly support radiogenomic prognostic prediction.

## 4 Recent Advances of Radiogenomics Studies in Human Diseases

**FIGURE 4:**
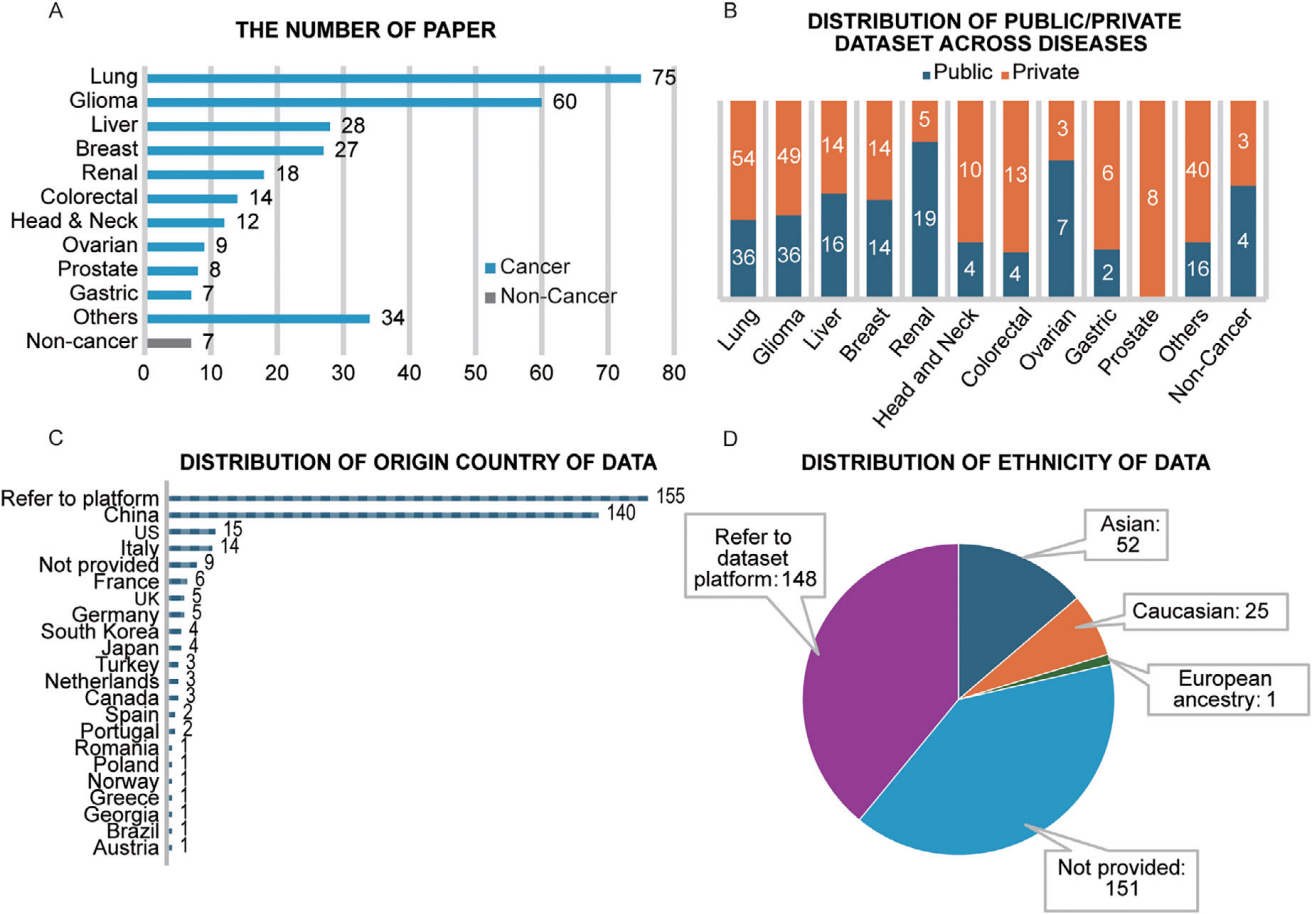
Landscape of recent rediogenomic studies. (A) Number of published papers by disease type, with oncological diseases represented by blue bars and nononcological diseases represented by gray bar. (B) Proportion of public versus private datasets by disease type, with public dataset represented by blue and private dataset represented by orange. (C) Distribution of dataset by origin country. (D) Distribution of ethnicities in radiogenomic studies. (Data presented are derived from articles in which the relevant information was available.)

The application of radiogenomics in human diseases encompasses oncology and nononcology diseases, with over 90% of studies focusing on oncology (Figure 4A). Relevant datasets were reported a total of 377 times (for data usage frequency). Public datasets accounted for 155 reported usages, with no public dataset reported for prostate cancer (Table 2). Public data availability for colorectal, gastric, and head‐and‐neck cancers were limited, with reporting rates below 30% (Figure 4B). Among private datasets, most frequently involved are from China, accounting for 139 times of reporting (Figure 4C). Although most private datasets did not specify the predominant ethnic composition, Asian, Caucasian, and European ancestries were explicitly mentioned in 52, 25, and 1 reported usage respectively (Figure 4D). In rest of this section, we systematically review radiogenomic advances according to disease type, highlighting the unique molecular‐imaging associations observed across different diseases.

**TABLE 2:** Summary of available public datasets for radiogenomic analysis.

Disease type	Project name	Platform	References^a^
Lung cancer	NSCLC radiogenomics	TCIA, TCGA	[97, 125, 126, 127, 128]
	TCGA_LUAD	TCIA, TCGA	[97, 126]
	TCGA_LUSC	TCIA, TCGA	[126, 129]
	CPTAC_LUAD	TCIA, TCGA	[126, 129]
	CPTAC_LSCC	TCIA, TCGA	[126]
	TCGA_LUSD	TCIA, TCGA	[97, 130]
	GSE103584	TCIA, GEO	[79, 109, 127, 131, 132]
Glioma	BraTS‐19	TCGA	[133]
	LGG‐1p19q deletion	TCIA	[134, 135]
	BraTS2021	BraTS official website	[136, 137]
	TCGA‐LGG	TCIA, TCGA	[138, 139, 140, 141, 142, 143, 144]
	TCGA‐GBM	TCIA, TCGA	[6, 94, 138, 139, 140, 142, 144]
	REM‐BRANDT	TCIA, GEO	[139, 145]
	UCSF‐PDGM	TCIA	[139, 146]
	UPENN‐GBM	TCIA, TCGA	[144]
	CPTAC‐GBM	TCGA	[6]
Liver cancer	TCGA‐LIHC	TCIA, TCGA	[8, 130, 147, 148, 149, 150, 151, 152, 153, 154]
	TCIA‐HCC‐TACE‐Seg	TCIA, TCGA	[152, 154]
Breast cancer	TCGA‐BRCA	TCIA, TCGA	[90, 98, 103, 110, 155, 156]
	I‐SPY2	TCIA, GEO	[157]
	GSE45827	GEO	[63]
	GSE129559	GEO	[63]
	GSE162228	GEO	[63]
	GSE81538	GEO	[63]
Renal cancer	TCGA‐KIRC	TCIA, TCGA	[70, 72, 73, 80, 81, 106, 130, 158, 159, 160, 161]
Esophageal carcinoma	TCGA‐ESCA	TCIA, TCGA	[162]
Colorectal cancer	TCGA‐COAD	TCIA, TCGA	[163, 164, 165]
	TCGA‐READ	TCIA, TCGA	[165]
Ovarian cancer	TCGA (OV)	TCIA, TCGA	[166, 167, 168, 169]
Gastric cancer	TCGA‐STAD	TCIA, TCGA	[170]
Soft tissue sarcoma	SARC021	SARC	[171]
Bladder cancer	TCGA‐BLCA	TCGA	[172]
Pancreatic cancer	CPTAC‐PDA	TCIA	[173]
Tuberculosis	Not available	TB portals	[174]

^a^ References listed some examples of studies employing the corresponding public dataset.

### 4.1 Cancer

#### 4.1.1 Lung Cancer

Lung cancer ranks the first in both global cancer incidence and mortality rate, posing a significant threat to public health [175]. Radiomics has been extensively utilized as a noninvasive approach for detecting key genetic mutations in lung cancer, successfully predicting the mutation status of critical driver genes such as EGFR [83, 96, 108, 114, 116, 176, 177, 178, 179, 180, 181, 182, 183, 184, 185, 186, 187, 188, 189, 190, 191, 192, 193, 194, 195, 196], TP53 [179, 183, 185, 186], T790M [85, 192, 195], ALK [191], KRAS [124, 131, 177, 179, 186, 190, 191], and others [197]; expression level of prognostic genes such as CD74 [109], ALOX5 [198], TACC3 [127], HOPX [199], and others [113, 200, 201]; genetic subtypes [125, 129, 202, 203, 204]; immune cell infiltration [205]; and tumor mutational burden (TMB) [117]. For instance, Li et al. retrospectively analyzed 2171 non‐small cell lung cancer (NSCLC) patients and demonstrated that a multidimensional model based on preoperative CT scans and clinical factors achieved an AUC of 0.843 in predicting EGFR/TP53 comutation status [183]. Hu et al. developed radiomic model using logistic regression based on contrast‐enhanced CT (CECT) scans to predict ALOX5 expression in NSCLC patients and achieved an AUC of 0.783. Patients with higher radiomic score shown significantly improved overall survival (OS), highlighting the potential of radiomics for noninvasive prognostic assessment in NSCLC [198]. These advances have provided valuable guidance for frontline clinical decision‐making, particularly in the selection of targeted therapies [183, 184, 198, 205].

Recent studies have increasingly focused on integrating quantitative imaging features with molecular data. This radiogenomic integration has led to the development of robust models that significantly enhance the precision of cancer management. For instance, combining radiomic signatures with genomic information has improved the ACC of diagnosis [7, 206]. Moving beyond diagnosis, these models have demonstrated strong performance in predicting complex clinical endpoints, such as metastasis [101, 207], mutation status [131], and response to therapy [208, 209, 210]. Furthermore, integrative models have been utilized to assess survival risk, providing individualized prognostic information that can inform clinical decision‐making and patient counseling [97, 120, 122, 132, 211, 212, 213, 214].

Research have also explored the relationship between radiomic features or radiomic subtypes and the underlying genetic variations and molecular pathways in lung cancer [79, 102, 126, 128, 215, 216, 217, 218, 219, 220, 221, 222, 223, 224, 225, 226]. For example, Dong et al. retrospectively analyzed 497 patients with clinical stage T1 lung adenocarcinoma (LUAD) and demonstrated that consensus clustering of CT radiomic features could stratify patients into two distinct risk groups, which were significantly associated with pathological risk factors and HER2 mutation status. [215]. In a multi‐institutional study of 394 NSCLC patients, researchers established a habitat imaging framework using pretreatment CT and PET. The identified imaging subtypes independently predicted disease recurrence beyond clinicopathological factors and ctDNA. Radiogenomic analysis revealed that the high‐risk subtype was characterized by downregulation of interferon alpha and gamma pathways. [128]. Collectively, these studies show the value of imaging‐based subtypes for personalized risk stratification and treatment planning in lung cancer.

#### 4.1.2 Glioma

Glioma is the most common primary intracranial tumor, accounting for approximately 80% of malignant brain tumors [227]. Molecular characterization has become increasingly important in glioma diagnosis and classification. The World Health Organisation 2021 Classification of Tumors of the Central Nervous System underscores the importance of IDH mutation and 1p/19q codeletion in glioma subtyping [228], as these genomic alterations are often associated with specific imaging patterns. For example, IDH‐mutant gliomas often show T2/FLAIR mismatch and lower perfusion on rCBV imaging, while 1p/19q‐codeleted gliomas are more likely to present with calcification and less frequent T2/FLAIR mismatch compared with noncodeleted tumors [229, 230, 231, 232]. Advanced radiogenomic analysis has enabled the noninvasive assessment of glioma subtypes using radiological images alone or in combination with patient clinical information [5, 133, 135, 142, 233, 234, 235, 236, 237, 238, 239]. Importantly, several predictive models have shown robust stratification performance in external validation cohorts, underscoring their potential clinical utility [134, 240, 241, 242, 243, 244, 245, 246].

Beyond established molecular markers in glioma subtyping, other genomic features also offer important implications for glioma treatment and prognosis, which are also frequently explored by radiogenomic studies. For instance, the presence of O6‐methylguanine‐DNA methyltransferase (MGMT) promoter methylation is associated with increased SEN to chemotherapy and improved outcomes in glioma patients. ML and DL models using MRI and PET features showed moderate ability in predicting MGMT promoter methylation status [5, 136, 137, 138, 146, 247, 248]. Notably, Zhu et al. reported comparable prediction ACC between the visual assessment of radiologists and the developed radiomic model [146]. In addition, variations in key driver genes implicating in glioma pathogenesis, such as TP53 [229, 249, 250], BRAF [251], ATRX [138, 230, 252], EGFR [229, 230, 245, 249, 253, 254], TERT promoter [5, 138, 229, 230, 241, 255, 256, 257], CDK6 [139], IL18 [143], and a range of other genes [141, 145, 258, 259, 260], as well as prognostic genomic signatures [133, 140, 261, 262, 263] have shown correlations with imaging features.

Integrative radiogenomic approaches have demonstrated strong predictive power for glioma patient outcomes. Combining DNA methylation with MRI‐derived Radscore can accurately predict recurrence risk and OS in glioblastoma [89, 94]. Models that integrate radiomic features with polygenic risk score, clinical data, or lncRNA/mRNA have achieved high ACC in survival prediction, comparable to biopsy‐confirmed molecular status [95, 121, 264].

Radigoenomic approaches have also contributed to the discovery of novel glioma subtypes. Imaging features can predict novel subtypes defined by transcriptional subclones and TME profiles, while unsupervised clustering of radiomic data has revealed imaging‐based subtypes with unique survival and genomic landscapes [6, 265]. Further, radiomic analysis of diffusion weighted imaging (DWI) images can distinguish immunesilencing and immuneactivating subtypes [144]. Notably, marker genes specific to immuneactivating subtype were found to be mutated exclusively within this group. DL model based on MRI has successfully predicted the survival of glioblastomas, with model outputs showing significant correlations with core signaling pathways and key genomic alterations [266, 267].

#### 4.1.3 Liver Cancer

Liver cancer remains one of the leading causes of cancer‐related morbidity and mortality worldwide, with hepatocellular carcinoma (HCC) accounting for the majority of primary liver malignancies. The clinical management of liver cancer is particularly challenging due to its pronounced molecular heterogeneity and the complexity of its TME. In recent years, radiogenomic studies in liver cancer, especially HCC, have increasingly focused on elucidating the associations between radiomic features or patient groups defined by radiomic features and TME components, biological pathways, and genes mutations [8, 78, 147, 148, 149, 268, 269, 270, 271]. For example, Xu et al. developed a multimodal fusion system that integrates CT‐derived DL features with clinical data to predict OS and progression‐free survival (PFS) in unresectable HCC patients receiving immune checkpoint inhibitors (ICIs). The developed model demonstrated superior prognostic performance compared with traditional radiomics and clinical benchmarks (C‐index: OS = 0.74, PFS = 0.69) and revealed enrichment of the PI3K/Akt pathway in high‐risk patients [8]. These findings provide mechanistic support for the use of imaging biomarkers to noninvasively capture the intricate pathophysiological states within HCC.

Furthermore, radiomics‐based signatures have been utilized to predict common molecular alterations in HCC, such as the gene expression level [150, 151, 153, 272, 273, 274, 275], mutational status of β‐catenin activation [276], TP53 [84], and RAS [76], and molecular subtypes [277, 278], which are closely linked to tumor behavior and patient prognosis. For example, researchers redefined the ICI‐responsive TME in HCC by integrating hypoxic scores and immune infiltrate abundance. They developed an immune hypoxic stress index that, together with MRI‐radiomic models, effectively stratified ICI responders (AUC > 0.80) and provided a clinically applicable approach for personalized immunotherapy [272]. Such noninvasive precise diagnosis facilitates risk stratification and personalized management of HCC patients.

In addition, recent studies have integrated radiomic features with genomic data (e.g. driver gene mutations, gene expression profiles) and clinical parameters (e.g. serum alpha‐fetoprotein levels and tumor staging) into multivariate predictive models [77, 88, 92, 93, 152, 154, 279]. For example, by integrating clinical information, mutational burden of key signaling pathways, and CT‐based semantic and radiomic features, researchers developed combined prognostic models for HCC patients treated with transarterial chemoembolization (TACE) and tyrosine kinase inhibitors (TKIs). The developed model achieved high predictive ACC for OS and PFS (C‐indices up to 0.805 and AUCs up to 0.917) [88]. These integrative approaches have demonstrated improved ACC in forecasting recurrence risk and OS in HCC patients, thereby supporting more precise clinical decision‐making and follow‐up strategies.

#### 4.1.4 Breast Cancer

Breast cancer is the most prevalent malignancy among women worldwide [175]. Advances in genomic characterization have substantially influenced patient stratification and prognosis assessment in breast cancer. In recent years, radiogenomics has emerged as a promising approach for noninvasive molecular profiling in breast cancer. Extensive efforts have identified unique imaging phenotypes from ultrasound and MRI associated with specific genomic variations [9, 10, 63, 280, 281]. Importantly, radiogenomic associations can vary significantly across different breast cancer subtypes, highlighting the need to explore radiogenomics within specific molecular subtypes rather than applying a one‐size‐fits‐all approach [10]. Building on association analysis, radiogenomic models have shown promise in the noninvasive detection of clinically relevant gene alterations, such as BRCA1/2 and TP53 mutations [155, 282, 283, 284, 285], HLA‐DQA1 and CCR5 expression [111, 156], as well as 21‐gene recurrence score and pathogenic mutation status [286, 287]. Notably, two large‐cohort studies (*n* > 400) have shown that combining radiomic features from both tumor and peri‐tumor regions and adding other clinical information can significantly enhance prediction ACC [282, 284].

Axillary lymph node (ALN) burden decides whether ALN dissection is needed for breast cancer patients. Current biopsy method to detect ALN positivity is invasive and carries risk for patients. To address this, radiomic features associated with ALN‐related genes have been selected to build models for noninvasively predicting ALN status, achieving comparable performance to gene‐based approach [110]. Furthermore, a previous study has reported upregulation of migration pathway and downregulation of cell differentiation pathway in image‐predicted high‐risk patients; common genes associated with radiomic and DL models were enriched in Ras, MAPK, P53 signaling pathways, offering novel biological insights into ALN metastasis [103]. Moreover, a nomogram incorporating both radiomics and genomics achieved AUC of 0.928 in predicting ALN metastasis, underscoring the value of multiomic models.

TME is a critical factor influencing tumor behavior and treatment response. Recent studies have demonstrated that specific imaging features and predictive models are associated with TME characteristics [157, 288, 289, 290]. Specifically, longest diameter, functional tumor volume, background parenchymal enhancement, and sphericity on breast dynamic contrast‐enhanced MRI (DCE‐MRI) are linked to pathological complete response (pCR) and hallmark pathways related to proliferation, immune, and signaling [290]. DCE‐MRI dynamic patterns predictive of pCR are related to differential expression in prognosis‐ and angiogenesis‐related genes as well as immune‐related pathways [157]. Furthermore, predictive imaging models have revealed distinct immune cell compositions in TME between high‐risk and low‐risk patients [288, 289]. Besides, radiological images also offer unique advantages in assessing tumor‐stromal heterogeneity by capturing the entire tumor and its surrounding tissues [90]. These findings highlight the critical role of radiogenomics in reflecting TME changes and its potential to guide immunotherapy.

#### 4.1.5 Renal Cancer

Radiogenomic research in clear cell renal cell carcinoma primarily centers on survival analysis, immune cell infiltration, and histological/nuclear grade prediction. Greco et al. identified imaging features associated with genes such as ADAM12, ADFP, and P4HA3, as well as OS [72, 73, 74]. Wang et al. developed a gradient boosting machine (GBM) model for FOXP3 prediction, finding that a higher Radscore was correlated with poorer OS [81]. He et al. used SVM to predict CTLA4 expression [106]. Chen et al. and He et al. constructed OS‐related risk scores based on pathway genes, classified patients using radiomic models, and integrated Radscore with clinical features to build nomograms for survival analysis, achieving high predictive performance (AUCs up to 0.948 and C‐index up to 0.807) [158, 291].

In terms of immune infiltration and grade prediction, Song et al. linked high immune infiltration to better anti‐PD‐1 therapy response and developed an ExtraTrees‐based radiomic model for its noninvasive prediction [80]. Greco et al. identified CT‐derived features for noninvasive GIMAP expression assessment [292], while Lv et al. and He et al. achieved good performance in histological and nuclear grade prediction (AUCs up to 0.94), though no universal association between radiomic features and nuclear grade‐related mRNA was found [66, 293]. Radiomic models have been used to predict the mutation status of genes such as TP53 [294], VSX1 [104], hypoxia‐related biomarkers [159], NCOA7 [64], and ADFP [70, 160]. Additionally, by linking imaging features with gene expression levels, these models offer insights into tumor growth and invasion patterns. Notably, Liu et al. demonstrated that stratifying patients by tumor grade or stage significantly improved the ACC of mutation prediction (AUC increased from 0.68 to 0.86) [160].

#### 4.1.6 Head and Neck Cancer

Radiogenomic research in head and neck cancer primarily focuses on squamous cell carcinoma (SCC) subtypes. In head and neck SCC, Wang et al., Zhu et al., and Spielvogel et al. developed radiomic models to predict key genes (FOXP3, CTLA4, IDH1) or radiomic‐genomic feature pairs, achieving AUCs of 0.582–0.948 in survival analysis [65, 67, 75, 82]; Li et al. integrated multiomic models for induction chemotherapy response prediction (AUC 0.88) [100], and Nguyen et al. realized noninvasive classification of immunotherapy‐related hot/cold tumor types via CT features [100]. In oral SCC, Kim et al. linked ASB2 overexpression to PET radiomic features like GLRLM_GLNU and poor targeted therapy response [69]. Corti et al. constructed SVM classifiers for immune‐related gene signatures [68], while Jin et al. associated the salivary hair bulge pathway and radiomic feature Original_glrlm_RunVariance with lymph node metastasis [71]. In oropharyngeal SCC, Li et al. achieved an AUC of 0.91 for HPV‐related P16 prediction [295], while Lyu et al. identified that neoadjuvant chemotherapy (NAC) poor responders featured enhanced keratinization and good responders had upregulated immune response or oxidative stress via radiogenomic analysis [296].

Beyond SCC, radiogenomic applications also extend to head and neck paragangliomas. A radiogenomic study provides novel insights into the correlation between PET imaging features and underpinning molecular dysregulation, expanding the scope of radiogenomic exploration in head and neck malignancies [297].

#### 4.1.7 Colorectal Cancer

Colorectal cancer (CRC) is the third most common cancer globally, accounting for 9.6 and 9.3% of global incidence and cancer‐related death respectively. Noninvasive prediction of gene expressions such as KRAS or BRAF enables timely prognostic assessment and treatment selection [175]. Radiogenomics has emerged as a pivotal tool in CRC management, leveraging radiomic features from diverse imaging modalities to predict key molecular alterations. For KRAS mutations prevalent in 30–50% of CRC cases and critical for guiding anti‐EGFR therapies, multiple robust predictive models have been developed, with overall AUCs ranging from 0.8‐0.9 [298, 299, 300, 301, 302, 303]. Wang et al. utilized venous‐phase CECT radiomics and XGBoost, where texture features reflected uneven enhancement tied to cell density and vascularity [298]. Zhang et al. fused arterial‐ and venous‐phase DL features and achieved better model over conventional radiomics. The higher prediction score was associated with poorer 5‐year survival, elevated carcinoembryonic antigen (CEA), and lower disease control [299]. In rectal cancer, Gan et al. utilized endorectal ultrasound radiomics from intratumoral and peritumoral regions to predict KRAS mutations [300, 303]. They obtained improved model performance by fusing radiomics and DL, where feature‐based fusion was superior to prediction‐based fusion. Lv et al. innovated multimodal fusion via DRMF‐PaRa, showing integrated pathomics and radiomics over unimodal baselines [301]. In contrast, Porto‐Alvarez et al. favored clinical nomograms over radiomics in CT scans, noting the potential of wavelet–Haralick features but no added value from imaging alone [302].

Saber et al. applied TabNet to preoperative CT radiomics in CRC liver metastasis, predicting CD73 expression, which may be a biomarker of prognosis and response to immunotherapies [304]. Kang et al. constructed CT‐based nomograms for BRAF mutations in 100 patients (training AUC 0.826), incorporating lymph node status and tumor features [305]. Zhong et al. used T2‐weighted MRI nomograms for p53 in rectal cancer, prioritizing wavelet–LLH skewness alongside clinical factors [306]. Ma et al. combined MRI radiomics (e.g. Imc1, Coarseness) with KRAS/CEA for rectal cancer liver metastasis prediction (combined AUC 0.842; KRAS odds ratio 8.296), underscoring mutations' independence for model prediction [307].

Multimodal paradigms shine in the diagnosis and prognosis prediction of CRC. CT radiomics has been applied in Stage II CRC patients and revealed AC‐preferable (Notch‐enriched) versus observation‐preferable (MYC/E2F) pathways (iHR 5.35) [163]. Additionally, CT radiomics outperformed PET in detecting recurrent rectal cancer [308]. DL‐radiomics fusion has shown promise in detecting microvascular invasion in colon cancer, effectively stratifying high‐risk groups with upregulated checkpoints [164]. Furthermore, the integration of imaging features and specific genes, such as INHBB and IL26‐related genes, has demonstrated potential in predicting 1/3/5‐year survival and metastasis, with AUCs exceeding 0.8 [165, 309].

#### 4.1.8 Ovarian Cancer

Ovarian cancer is usually diagnosed at an advanced stage (Stage III–IV) due to nonspecific symptoms in the abdomen and pelvic region, with up to one‐fourth of the cases can be attributed to hereditary factors, such as BRCA1/2 gene mutation. Although surgical techniques and other novel treatment modalities such as targeted therapy have been optimized, the 5‐year OS remains poor, ranging between 10 and 40% [310]. Similar to other cancers, radiogenomics has become a promising approach for linking imaging phenotypes with molecular alterations and prognosis variations in ovarian cancer patients, thus improving choice of treatment strategy. CT radiomics has shown potential in predicting the expression of genes, such as CSF3 [311], CXCL10 [166], CXCL13 [167], CCR5 [168], and BRCA [312], which further correlate with survival. Kishi et al. identified nine prognostic CT‐based radiomic biomarkers in serous ovarian cancer (SOC) related to G2M checkpoints, mitotic spindle and E2F targets, all of which are influential to cell cycle regulation and proliferation [169]. These biomarkers stratified patients into groups enriched for cell cycle pathways. Similarly, key features in prognostic model of OS shown association with EMP1 expression [313]. Crispin‐Ortuzar et al. further advanced the field by integrating multisite CT radiomics with ctDNA metrics, such as TP53 mutant allele fraction and trimmed median absolute deviation, to predict response to NAC [314]. The combined radiogenomic model achieved an AUC of 0.78. Notably, Ju et al. conducted a pioneering study integrating spatial transcriptomics with CT phenotypes in high‐grade SOC [315]. Imaging features such as bilaterality, nodular seeding, and parietal peritoneal thickening were associated with pathways of poor prognosis, including TNF‐α/NF‐κB, oxidative phosphorylation, and E2F/MYC targets. Conversely, chemotaxis and immune‐modulatory genes (CXCL14, NTN4, DAPL1, RNASE1) characterized favorable phenotypes. Despite its limited sample size, this study provided the first spatially resolved demonstration of radiogenomic heterogeneity in high‐grade SOC.

#### 4.1.9 Gastric Cancer

Gastric cancer (GC) is the fifth most common cancer and fourth leading cause of cancer death worldwide [316]. Radiogenomics in GC links CT‐derived radiomic features with corresponding molecular and immune profiles, offering novel biomarkers for classification, prognosis prediction, and therapy guidance. In molecular diagnosis, venous‐phase CECT can reflect homologous recombination genes RAD51D and XRCC2, showing potential to guide chemotherapy decisions [317]. Similarly, arterial‐ and venous‐phase CECT radiomics can robustly predict KIT exon 9 mutation in gastrointestinal stromal tumors (GIST), underscoring the generalizability of combined multiphase imaging [318]. For prognosis prediction, Nath et al. combined CT, gene expression, and clinical data and identified that age, CSF1R, CXCL12, and texture metrics are important survival predictors [170].

Radiogenomic studies have also linked imaging features of GC to immune markers and immunotherapy responses. Dai et al. developed a light GBM model to predict PD‐L1 expression from wavelet features, highlighting relevance between multiscale image texture and immune checkpoint expression [319]. Zhan et al. identified a nine‐feature signature that discriminates microsatellite instability (MSI)‐high tumors correlated with immunotherapy response, which suggested the more complex but uniform gray‐level textures in MSI‐high tumors [320]. Additionally, Yang et al. connected CT‐based extramural venous invasion (EMVI) score with EMVI‐related gene signature, showing that the group with high EMVI score exhibited lower MSI or TMB and poorer response to ICIs [321, 322]. It is also noted that GC is a highly inflammatory‐mediated cancer with high heterogeneity regarding the inflammatory microenvironment, such as M2 macrophages infiltration.

#### 4.1.10 Prostate Cancer

Prostate cancer is ranked globally the most common cancer among men, reaching around 1.5 million new cases and 375,000 deaths annually [316]. Radiogenomic studies in prostate cancer have linked imaging features to several key driver genes. MR functional imaging such as DWI and multiparametric MRI has revealed associations with proliferation‐related gene expression and angiogenic transcriptional activity, highlighting the biological relevance of functional imaging [323, 324]. Texture features from MRI have been linked to hypoxia‐ and angiogenesis‐related genes such as ANGPTL4 and P4HA1, with increased radiomic textural heterogeneity indicating more aggressive, hypoxic tumors and poorer prognosis [325, 326]. Noninvasive detection of FOXA1 mutations cannot be reliably detected by current clinical parameters such as T stage, Gleason score or metastasis status, but an MRI based radiomic model with highest performance of AUC over 0.8 using random forest classifiers [327]. Detection of multiple pathogenic DNA damage repair gene mutations such as BRCA1/2, CDK12, and ATM genes can be achieved by using MRI‐based radiomic models with AUCs above 0.8, which can theoretically reduce unnecessary genetic testing by 25% [328]. Beyond MRI, the integration of B‐mode ultrasound, contrast‐enhanced ultrasound, and transcriptomic profiling improved the diagnostic ACC of prostate cancer versus benign prostatic hyperplasia, where the radiomic texture and perfusion features shown correlations with genes involved in regulating lipid metabolism, epithelial–mesenchymal transition, and androgen resistance [329].

#### 4.1.11 Others

Beyond the extensive studies in common cancers, recent studies have also highlighted the significant value of radiogenomics in a wide range of other malignancies. Radiogenomic analysis have revealed associations between imaging features and genomic expression or pathways in esophageal cancer [115, 119, 162], pancreatic cancer [173, 330], and retinoblastoma [331]. Notably, in diffuse large B‐cell lymphoma, radiomic features correlated with metabolic gene signature have enabled more precise prognosis stratification [332]. Clustering of multisequence MRI features in skull base chordomas has revealed survival differences linked homozygous 9p21 deletions and 1p36 deletions [333]. However, insignificant association has been reported between pancreatic cancer and XRCC1 polymorphism [334].

Radiomic and DL models based on MRI, CT, and ultrasound have shown promising results in predicting molecular subtypes and genetic alterations across multiple cancer types. These include NF‐2 mutation in meningioma [335], KIT 11 mutation in GIST [336], angiogenesis‐related genomic risk score in bladder cancer [172], MDM2 amplification in retroperitoneal well‐differentiated liposarcomas and lipomas [337, 338], EGFR mutation in brain adenocarcinoma [339], TP53 and MYCN alterations in endometrial cancer and neuroblastoma [340, 341], immunology subgroups in cholangiocarcinoma [330], as well as several key gene alterations in intrahepatic cholangiocarcinoma and papillary thyroid carcinoma [342, 343].

In addition to static associations, longitudinal radiogenomic analysis provides insights into tumor evolution and treatment response. Delta‐radiomics derived from sequential pretreatment MRI scans of soft‐tissue sarcoma have been shown to reflect the natural evolution of the tumor, with feature clustering identified a patient group characterized by poor outcome and overexpression of Hedgehog pathway genes [344]. For multimetastatic soft tissue sarcoma, intrapatient radiomic variability on CT images has been shown to moderately correlate with posttreatment ctDNA positivity, aiding the identification of treatment‐resistant patients [171]. Likewise, changes in SUVmax on PET imaging have demonstrated strong correlation with changes in ctDNA before and after treatment, suggesting its potential as biomarkers for treatment response surveillance [345].

The integration of imaging features and various genomic data has demonstrated the value of radiogenomics models in clinical tasks, including diagnosis [346], improved staging [347], metastasis prediction [348], and survival prediction [119, 349, 350, 351, 352]. Moreover, several studies have suggested that the combined radiogenomics models outperformed separate genomic or radiomic model, highlighting the generalized improved value of combining multiomic information [119, 347, 348].

Unlike previous studies focused on single cancer type, pan‐cancer research has demonstrated the generalizability of radiogenomic analysis. Sangeetha et al. shown the improved early detection of multiple cancer types using transformer‐based multimodal models [353]. Italiano et al. revealed the predictive ability of CT‐radiomics in predicting key driver gene variations in pan‐cancer [354]. Additionally, Bernatowicz et al. utilized CT‐radiomics to classify T‐cell‐inflamed versus T‐cell‐uninflamed patients in pan‐cancer cohorts and to monitor immune status transitions during immunotherapy, providing valuable complementary tool for evaluating immunotherapy treatment effect [355].

### 4.2 Non‐cancer

Radiogenomics has emerged as a powerful tool for elucidating disease mechanisms beyond oncology, particularly in neurodegenerative, infectious, and inflammatory conditions. In Alzheimer's disease, Jiang et al. employed radiomic features from 246 Brainnetome Atlas regions to construct a radiomics‐based structural covariance network (R2SN) and identified three neuropsychiatric symptoms (NPS) subtypes with distinct R2SN patterns by correlating regional gray matter volume variability with Allen Human Brain Atlas gene expression data [356]. Additionally, hippocampal radiomic features are associated with various biological functions in Alzheimer's disease, as evidenced by Xia et al., in which a T1‐weighted MRI radiomic model with 12 features was constructed [357]. Subsequent radiogenomics mapping also demonstrated enrichment of myeloid leukocyte and neutrophil activation, in addition to identification of six hub genes. Zhang et al. also derived a novel multitask genotype‐protein interaction and correlation disentangling method to unveil correlation patterns between genotype–protein interactions that contribute to MRI phenotypes of the brain, at a reduced computational power [358].

Shifting to respiratory diseases, Bui et al. combined chest X‐ray semantic features with Mycobacterium tuberculosis genomic SNPs for TB drug resistance analysis, achieving 92–94% ACC in distinguishing resistant from sensitive strain [174]. Xia et al. extracted 12 LoG‐filtered CT texture features correlated with ACE2 expression alterations to classify COVID‐19, LUAD, and critical illness [359]. More recently, Liu et al. constructed a thoracic CT radiomic model, classifying individuals into three risk groups, and a positive group of pneumoconiosis. TFCP2 is identified as a significant biomarker, positively correlated with pneumoconiosis risk [360].

In inflammatory bowel disease, Zhang et al. extracted radiomic features from multiparametric brain MRI and correlated with 137 blood metabolites and fecal 16S rRNA gut microbiota. R2^*^ value of the left hippocampus appears as the most distinguishing factor among 13 MRI radiomic features of the brain, as it attained the highest mean SHAP value. Subsequent mediation analysis revealed that microbiota may influenced blood flow in the brain via metabolites like TAG and phosphatidylinositol [361].

Across these studies, mediation analysis shows significant values to further explaining the relationships, in addition to GESA and simple correlation analysis, between genetic factors and imaging phenotypes, such as between radiomic features and molecular pathways. The outstanding predictive performance underscores radiogenomics’ versatility for noncancer precision diagnostics and biological mechanistic insights.

## 5 Discussion

Following the comprehensive literature review of recent radiogenomic studies in previous sections, this section offers an in‐depth discussion on how radiogenomics contributes to solving clinical problems, the current challenges in its clinical application, and the future directions for its development.

### 5.1 Clinical Problem‐Solving Radiogenomics

Although radiogenomics remains in its early stages in clinical translation, it has demonstrated considerable potential across various clinical applications.

#### 5.1.1 Deciphering Disease Biology and Heterogeneity

A central focus of radiogenomics research is the investigation of the intricate associations between imaging phenotypes and genomic alterations, aiming to advance the understanding of disease initiation, progression, and heterogeneity through integrated multiomics perspectives. On the one hand, a substantial body of studies have focused on validating and deciphering the imaging correlations of well‐recognized genomic features, such as key driver gene mutations and established genetic subtypes in diverse diseases [229, 230, 231, 280]. These investigations aim to identify the imaging characteristics that are spatially and quantitatively linked to specific genomic alterations, thereby laying a critical foundation for noninvasive genetic status assessment in clinical practice. On the other hand, the advent of high‐throughput sequencing technologies has facilitated a shift toward large‐scale radiogenomic association analysis. By integrating high‐dimensional imaging features with comprehensive gene expression profiles, these data‐driven approaches overcome the limitations of hypothesis‐driven research. As a result, they enable the identification of previously rarely recognized genetic markers and their imaging correlations [105, 171, 264].

#### 5.1.2 Enabling Noninvasive Genotyping and Dynamic Monitoring

The most extensively explored clinical application of radiogenomics is the prediction of genotypes using routine imaging examinations and analysis. Traditional genetic testing methods, which rely on tissue sampling via surgery or biopsy, are invasive and associated with inherent risks. Numerous studies have demonstrated that imaging‐based genotype prediction can serve as a noninvasive alternative, allowing clinicians to rapidly and safely obtain key molecular information from radiological images alone [96, 116, 177, 178, 180, 181, 182, 183, 336, 339, 340,343]. More importantly, as tumor molecular characteristics and TME may evolve during treatment, serial imaging examinations during the course of treatment enable physicians to dynamically monitor disease progression and therapeutic response [90, 344].

#### 5.1.3 Improving Prognostic Stratification With Multiomic Data

The integration of radiomics and genomics for prognosis prediction represents another pivotal advancement in the field of radiogenomics, offering significant clinical utilities with more accurate prognostic assessment. While genomic data provide precise molecular alterations within selected tissue samples, radiomic data offer complementary information regarding tumor morphology, heterogeneity, and microenvironment. By combining macroscopic imaging phenotypes with microscopic genetic characteristics, the multiomic approach effectively addresses the limitations of single‐modal prognostic models. Many previous studies have suggested that radiogenomics prognostic models outperformed those based solely on radiomic or genomic data, underscoring the value of combining multimodal data [87, 89, 91, 94, 95, 121, 346, 347, 348, 349].

#### 5.1.4 Facilitating Treatment Decision‐making

Building upon the aforementioned applications, the ultimate goal of radiogenomics is to facilitate treatment decision‐making. It demonstrates significant potential across three key stages. First, the multiperspective insights into disease pathogenesis can aid in uncovering novel disease subtypes, thereby informing the discovery of new therapeutic targets and development of novel drugs [105]. Second, the noninvasive detection of genotypes enhances the feasibility of personalized treatment in a safer and cheaper way, particularly for patients who may not have access to genetic testing. The obtained molecular information can be leveraged to tailor treatment regimens to individual patients, thereby minimizing ineffective therapies and enhancing treatment efficacy. Additionally, the capacity of dynamic, noninvasive monitoring of genetic or TME alterations during treatment allows active surveillance and timely adjustment of treatment strategies [345]. Third, the improved long‐term prognostic stratification, such as survival and treatment resistance, provides critical information for the doctors to guide the selection of appropriate treatment approaches, whether more aggressive or conservative interventions are warranted [119, 174].

### 5.2 Challenges

Despite significant advances in radiogenomics for molecular subtyping, prognostic prediction, and therapeutic guidance in cancer, the clinical translation of this field faces multiple challenges.

#### 5.2.1 Scarcity of High‐Quality Radiogenomic Data

A significant challenge impeding the advancement of radiogenomics is the scarcity of high‐quality, paired radiogenomic datasets. Unlike radiological examinations that have become routine in clinical practice, the high price of gene tests restricts its widespread usage, making paired radiogenomic data hard to obtain. Frequently cited limitations in radiogenomics studies, such as “small sample size,” “retrospective study design,” and “absence of external validation,” underscore the extent to which data scarcity constraints the generalizability and impact of research findings in this field. In addition, data standardization remains a persistent challenge in radiogenomic studies. The extraction and calculation of imaging features are substantially affected by several factors. These include the type and manufacturer of scanners, specific acquisition parameter and reconstruction settings employed, interradiologist delineation variations for ROIs, and considerable variability in image pre‐ and postprocessing procedures. Similarly, acquisition of genomic features is subject to substantial variability arising from differences in tissue procurement and preservation methods, the choice of sequencing platforms, and quality control and data preprocessing pipelined implemented. Furthermore, additional sources of variations are introduced during the integration of radiomic and genomic data, where diverse data processing workflows and artificial intelligence models can lead to divergent results even on the same dataset.

#### 5.2.2 Lack of Specialized Radiogenomic Analysis Method

The lack of specialized analytical methods tailored to address the complex, high dimensional, and multimodal nature of radiogenomic data represents a significant challenge to progress in the field. Existing analytical approaches are predominantly adapted from either radiomics or genomics, which were originally developed for single‐modality data analysis and cannot fully accommodate the unique characteristics of multimodality data. These methods mostly focus on one‐to‐one or one‐to‐many associations, necessitating the preselection of imaging features or genes of interest, which potentially introduces bias and limits the scope of discovery.

#### 5.2.3 Translation From Research to Clinic

Despite the promising findings reported in radiogenomics studies, there is still significant gap between research advancements and their translation into clinical practice. The first challenge in bridging this gap is the lack of rigorous validation of radiogenomic findings, particularly through animal experiments and prospective clinical trials. Second, there is high computational demand for the process and integration of high‐dimensional multimodal data, especially when combined with advanced large‐scale artificial intelligent models. Third, the integration of radiogenomic analyses into existing healthcare systems poses practical and organizational challenges, including the need for standardized workflow, interoperation with clinical information system, clinician training, and the special consideration of privacy problem.

### 5.3 Future Perspectives

#### 5.3.1 Data Availability, Standardization, and Reporting Guidelines

The current scarcity in public dataset warrants further planning in data dissemination. Having large and richly annotated datasets encompassing a broad spectrum of patients would drastically enhance the feasibility of producing generalizable models and robust external validation. Although fully accessible data may arouse privacy and security concerns, policy driven governed access could be a solution. With more public data available, the cost of data accrual and preparation at institutional level may be substantially reduced, allowing less resourceful institutions to leverage on large datasets combined with local data. Furthermore, currently reported datasets are largely from China, European countries and Northern America. Diversity and representativeness of data could be improved, for instance by developing policies and devoting more locoregional resources to curating datasets involving targeted ethnic groups.

To enhance comparability and transparency of radiogenomic research, the development and adoption of standardized protocols and reporting guideline for each step in the acquisition and analysis of data are urgently needed. Initiatives such as IBSI for radiomics and MIAME for genomics; and reporting guidelines such as RQS and CLEAR provide valuable frameworks. However, further harmonization and widespread implementation are essential for advancing the field.

#### 5.3.2 Multimodal Integration and Spatial Omics

Future research will increasingly move beyond single‐modality analysis toward cross‐scale, multimodal integration. In particular, upcoming studies will incorporate data from intermediate molecular pathways such as proteomics and metabolomics. These modalities act as essential functional bridges: proteomics identifies the effector proteins that execute gene transcription and regulate cellular architecture and function, while metabolomics offers information on the cellular energy state and microenvironmental processes. By integrating proteomic and metabolomic data with genomic profiles and single‐cell sequencing, researchers can dynamically track tumor clonal evolution and more accurately delineate the functional mechanisms related to drug resistance. In addition, spatial omics that captures the location of molecular distributions within tissues also complements current methods that treat tissues as homogeneously mixed. The comprehensive information may enable a deeper understanding of how molecular changes at different biological scales interact, and ultimately contribute to changes on radiological images.

#### 5.3.3 Specialized Analytical Methods and Platforms

The development of specialized analytical methods tailored for high‐dimensional radiogenomic data is essential for advancing the field. Innovative approaches, such as mapping high‐dimensional radiomic and genomic feature vectors into image‐like representations, can leverage DL networks to deeply explore the spatial and structural associations between imaging and genetic data. Alternatively, projecting these complex datasets into unique latent spaces may facilitate the integration and complementarity of multimodal information, enabling the discovery of novel and clinically relevant patterns. Furthermore, the establishment of an open and robust analytical platform will play a pivotal role in accelerating the evaluation and improvement of existing methods, as well as fostering the rapid iteration and dissemination of new analytical strategies. Similar to how data platforms like TCGA and TCIA have greatly expanded the scope of radiogenomic research by providing accessible genomic and imaging datasets, a shared platform for analytical methods would standardize and streamline the analysis process, enhance the reliability and reproducibility of research findings, and ultimately promote the clinical translation of radiogenomic discoveries.

#### 5.3.4 In Vivo Experiments and Clinical Trials

The clinical translation radiogenomic findings will critically depend on rigorous validation in both in vivo experiments and clinical trials. Future studies will move beyond in vitro data analysis and utilize in vivo systems such as patient‐derived xenograft (PDX) models or orthotopic genetically engineered mouse models to establish causal mechanisms between imaging features and specific genetic pathways [362, 363]. For example, researchers can observe whether certain imaging traits consistently appear in PDX models with defined mutations, and use gene editing (e.g., CRISPR) or pharmacological interventions to confirm that changes in imaging features correspond to molecular alterations. Future efforts should also prioritize prospective, multicenter, high‐quality clinical trials to validate the predictive reliability of radiogenomic models for treatment response and prognosis in real‐world settings. Demonstrating the clinical benefit and cost effectiveness of these models, such as improved patient outcomes (OS/PFS) or reduced overall treatment costs, will promote their widespread adoption.

## 6 Conclusion

Radiogenomics has been applied in both oncological and nononcological diseases, with the majority of studies concentrated in oncology. Although radiogenomics has yielded encouraging findings across various diseases, most findings remain preliminary and require further validation. Future research should focus on assembling shared large datasets, developing common platforms for data analysis, and conducting animal studies and prospective clinical trials to validate existing conclusions.

## Author Contributions

Xinyu Zhang, Qingpei Lai, Jin Cao, and Jerry Chi Fung Ching: conceptualization, investigation, data analysis, methodology, writing – original draft, and writing – review and editing. Xinzhi Teng: conceptualization, methodology, and writing – review and editing. Jiang Zhang: methodology and writing – review and editing. Shara Wee Yee Lee: supervision and writing – review and editing. Ge Ren: supervision and writing – review and editing. Jing Cai: conceptualization, funding acquisition, supervision, and writing – review and editing. All authors have read and approved the final manuscript.

## Funding

This study was partly supported by research grants of Projects of RISA (P0043001) and Projects of RI‐IWEAR (P0049375) from The Hong Kong Polytechnic University and Health and Medical Research Fund (HMRF 09200576), the Health Bureau, The Government of The Hong Kong Special Administrative Region.

## Ethics Statement

The authors have nothing to report.

## Conflicts of Interest

The authors declare no conflicts of interest.

## Supporting information




**Figure S1**. Literature search and selection process. Radiogenomic studies published as English full‐text articles within the past 3 years were search on three databases: Web of Science, PubMed, IEEE. Initial screening was performed based on abstracts, excluding review articles, conference papers, duplicates, and studies involving only radiomics or genomics. The remaining papers underwent full‐text screening to confirm the inclusion of radiogenomic analysis.

## Data Availability

The authors have nothing to report.
