# Supplementary material for: Radiogenomics: Current Understandings and Future Perspectives
Source: MedComm (2020). 2026 Jan 22;7(2):e70583. doi: 10.1002/mco2.70583 (PMC12828074; doi:10.1002/mco2.70583)

**Radiogenomics: Current Understandings and Future Perspectives**

**short title: A Review of Radiogenomics in Human Diseases**

Xinyu Zhang, MSc^1#^, Qingpei Lai, MSc^1#^, Jin Cao, MSc^1#^, Jerry Chi Fung Ching, BSc^1#^, Xinzhi Teng, PhD^1^, Jiang Zhang, PhD^1^, Shara Wee Yee Lee, PhD^1^, Ge Ren, PhD^1^, Jing Cai, PhD^1,2*^

1. Department of Health Technology and Informatics, The Hong Kong Polytechnic University, Hong Kong, China
2. The Hong Kong Polytechnic University Shenzhen Research Institute, Shenzhen, China

#These authors contributed equally to this work.

***Corresponding Author:**

Jing Cai: jing.cai@polyu.edu.hk

Figure S1. Literature search and selection process. Radiogenomic studies published as English full-text articles within the past three years were search on three databases: Web of Science, PubMed, IEEE. Initial screening was performed based on abstracts, excluding review articles, conference papers, duplicates, and studies involving only radiomics or genomics. The remaining papers underwent full-text screening to confirm the inclusion of radiogenomic analysis.


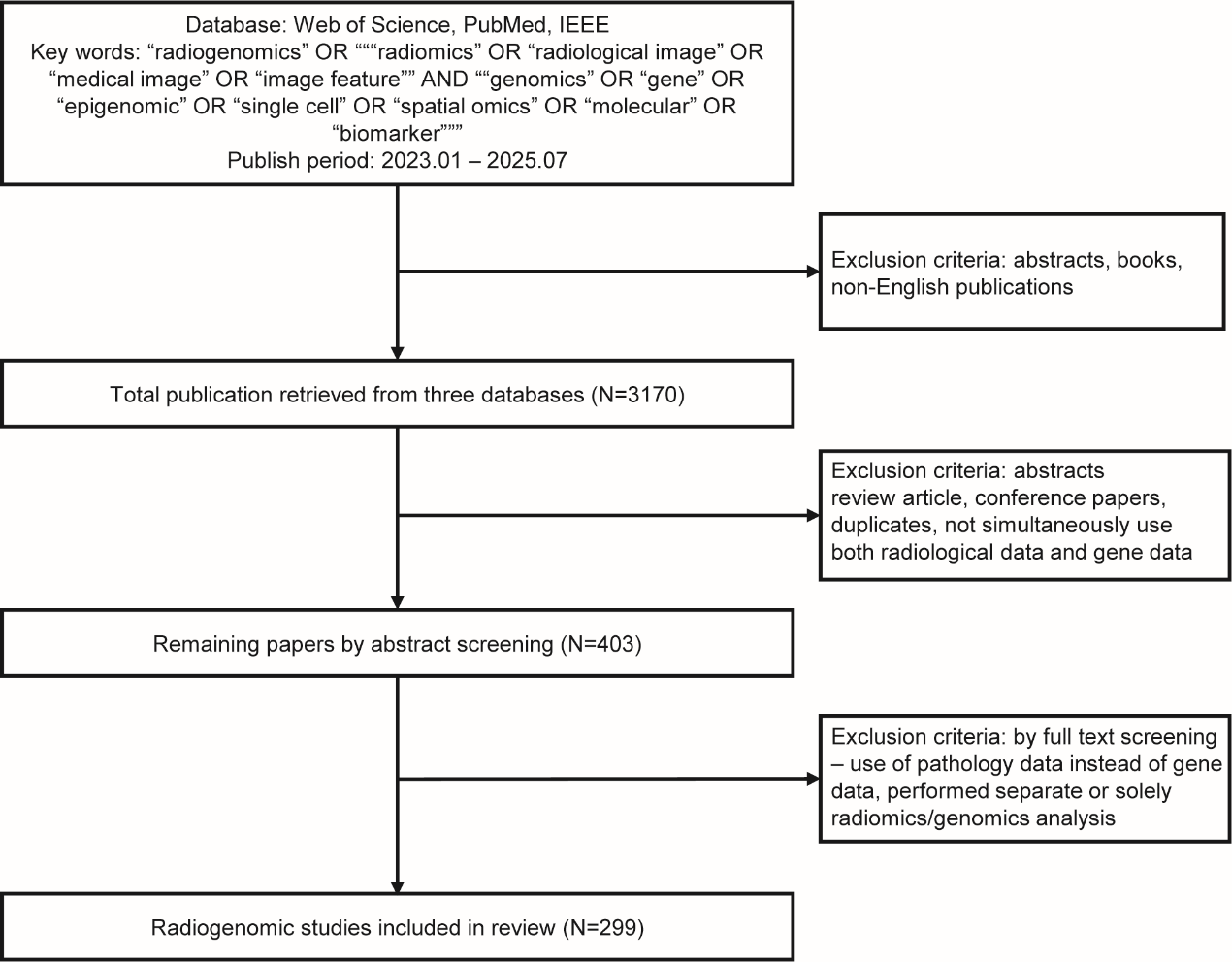

Supplement: Supplementary file 1 — Figure S1. Literature search and selection process. Radiogenomic studies published as English full‐text articles within the past 3 years were search on three databases: Web of Science, PubMed, IEEE. Initial screening was performed based on abstracts, excluding review articles, conference papers, duplicates, and studies involving only radiomics or genomics. The remaining papers underwent full‐text screening to confirm the inclusion of radiogenomic analysis. [file MCO2-7-e70583-s001.docx]
